# Dendrite Morphology Evolution of Al_6_Mn Phase in Suction Casting Al–Mn Alloys

**DOI:** 10.3390/ma13102388

**Published:** 2020-05-22

**Authors:** Zhongwei Chen, Yue Hou, Bin Xie, Qi Zhang

**Affiliations:** 1 State Key Laboratory of Solidification Processing, Northwestern Polytechnical University, Xi’an 710072, China; yueh0903@mail.nwpu.edu.cn (Y.H.); 2017200755@mail.nwpu.edu.cn (Q.Z.); 2 Wuhan BOE Optoelectronics Technology Co., Ltd., No.691 Linkonggang Road, Dongxihu District, Wuhan 430040, China; binxie@boe.com.cn

**Keywords:** Al–Mn alloys, suction casting, dendrite morphology, icosahedral quasicrystal

## Abstract

The effects of solute element content and cooling rate on the morphology of Al_6_Mn phase in suction casting Al–Mn alloys were investigated by transmission electron microscope, scanning electron microscope, and X-ray diffractometer. Results show that Al_6_Mn dendrite morphology with different degrees of development can occur in the microstructure of as-cast Al–Mn alloys. For the Al–4 wt.% Mn alloy, there are small amounts of block Al_6_Mn crystals at the center of sample, while we see a block Al_6_Mn phase and a feathery Al_6_Mn phase in the sample of Al–6 wt.% Mn alloy. Moreover, the block Al_6_Mn phases in the Al–8 wt.% Mn alloy disappear, and only snowflake-like Al_6_Mn phases play a dominant role in the microstructure. However, with an increase in Mn content to 10 wt.%, more dendritic trunks are formed, and secondary dendrite arms are degraded more seriously due to the formation of an icosahedral quasicrystal in suction casting. In addition to the effect of Mn content on Al_6_Mn morphology, with the increase in cooling rate from the center to the edge of samples, the outline diameter of equiaxed dendrite decreases. The evolution of Al_6_Mn dendrite morphology and the formation of quasicrystal are further discussed.

## 1. Introduction

As we all know, for the alloys with excellent mechanical properties, a good solidification structure is essential. According to the strict control of the microstructure formed, the mechanical properties of aluminum alloys can be enhanced by up to 20% during the casting process [1]. Critical alloying elements in commercial aluminum alloys are mainly transition metals (Mn, Cr, Fe, and the like), which were used for many years. Al–Mn alloy with an Mn content up to 1.5 wt.% has sufficient strength, good corrosion resistance, and excellent formability and weldability, allowing it to become the first choice of packaging and architectural application [2]. Notably, the Mn element content of widely used Al–Mn alloys in industry is generally between 1.0 wt.% and 1.6 wt.%, mainly because the solid solubility of Mn in α-Al is only 1.8 wt.% at 65 °C [3]. Furthermore, with the increase in Mn content, the continuous strengthening results in loss of toughness of Al–Mn alloys. Thus, looking for an easy way to amend such a conflict of strength and ductility remains a key issue.

Shechtman first discovered the icosahedral quasicrystalline phase (I-phase) in rapid solidification of Al–Mn alloys in 1984 [4], which attracted tremendous research attention regarding quasicrystal-strengthened Al–Mn alloy systems at high cooling rates of 10^4^–10^9^ K/s [5,6]. Although supersaturated solid solutions and quasicrystal phases can be formed in Al–Mn alloys during rapid solidification, in order to obtain bulk materials, the chemical composition of the alloy can be modified and the cooling rate required for I-phase formation can be effectively reduced. Juarez et al. [7] revealed that an I-phase at intermediate cooling rates of ~150 K/s can be obtained in aluminum alloys containing 8 at.% and 10 at.% Mn. Song et al. [8] added Be to Al–Mn alloy to successfully enhance the quasicrystal-forming ability. Other works [9,10] suggested that Be addition to the Al–Mn alloy influences a reduction in not only the critical amount of Mn element required to form quasicrystalline phases, but also the critical cooling rate for I-phase formation. Inoue et al. [11] obtained a quasicrystalline structure alloy with high tensile strength and good bending ductility by adding transitional elements to the Al–Mn alloy. Vojtěch et al. [12] studied the effect of the Sr element on the microstructure evolution of the Al–Mn alloy, and they found that Sr addition led to a reduction in the volume fraction of the I-Al_6_Mn phase. Jun et al. [13] investigated the effect of the addition of mish-metal on the formation of the I-phase in 94Al–6Mn samples and examined the samples with good mechanical properties, which were prepared by injection-casting into a copper mold. Kamaeva et al. [14] studied the crystallization sequence and the conditions of solid phase formation in slowly solidified Al–Cu–Fe alloys, where they observed that the I-phase forms from the melt, while the temperature of the melts before cooling determined the undercoolability and the crystallization characteristics in the concentration region. Chen [15] et al. found that many intermetallic compounds can potentially form in A3003 aluminum alloys, such as Al_6_Mn, and they demonstrated that the phase formation depends on not only the metallurgical history but also the actual alloy composition.

In fact, under equilibrium conditions, Mn concentrations up to 22 at.% can be achieved at the Al-rich side on the basis of the Al–Mn phase diagram. In the vicinity of face-centered cubic α-Al, several intermetallic phases can be formed [16]. These phases are an orthorhombic Al_6_Mn phase and two types of hexagonal approximants with structures closely related to quasicrystals: μ-Al_4_Mn and λ-Al_4_Mn. Markoli et al. [17] examined I-phase formation containing both Fe and Cu in Al-rich Al–Mn alloys, casted into a copper mold, which suggested that Fe and Cu elements can support the nucleation of I-phase. At intermediate cooling rates of 10^1^–10^3^ K/s, adding Fe to the 94Al–6Mn binary alloy induced the formation of a quasicrystalline phase and various metastable phases, as well as decagonal and icosahedral quasicrystals and their approximants [18,19].

The Al_6_Mn phase is known as the most aluminum-rich intermetallic compound in the Al–Mn system [20,21]. When the Mn element content is at a certain level in Al–Mn alloys, the dissolved solute content increases, resulting in the precipitation of the Al_6_Mn equilibrium intermetallic phase. Many researchers studied the orientation relationship between the Al_6_Mn phase and matrix [22,23], as well as the effects of Mn addition on the dispersoid formation behavior [24]. However, in order to obtain excellent mechanical properties of Al-based alloys for commercial applications with less severe cooling conditions for casting, efforts are being made to obtain quasicrystalline particles embedded in an α-Al matrix and a three-phase microstructure comprising the Al_6_Mn intermetallic phase. The aim of this work is to examine the effects of both cooling rate and solute content on the morphology of the Al_6_Mn intermetallic phase and the formation of a quasicrystalline phase. Particular attention is paid to characterizing the evolution of the micro-morphology at intermediate cooling rates of 10^2^–10^3^ K/s in suction casting.

## 2. Materials and Methods

Ingots of nominal composition Al–1.8 wt.% Mn, Al–4.0 wt.% Mn, Al–6.0 wt.% Mn, Al–8 wt.% Mn, and Al–10 wt.% Mn alloys were prepared by arc-melting a mixture of pure aluminum ingot (99.99 wt.%) and Al–20 wt.% Mn master alloy in a water-cooled copper crucible filled with argon. Cylindrical samples with a diameter of 4 mm and a length of 15 mm were obtained by vacuum suction casting, in which the cooling rate of the ingots ranged from ~10^2^ K/s (at the edge) to ~10^3^ K/s (at the center) during the casting process. The crystal structure was identified by an X-ray diffractometer (XRD, X′Pert PRO MPD, Almemo, Holland) with Cu K_α_ radiation operating at 40 kV/35 mA and diffractions in the 2θ range from 25° to 80°. The microstructure and the distributions of the chemical composition of these samples were studied with a scanning electron microscope (SEM, ZEISS-SUPRA 55, Jena, Germany) equipped with an attached X-ray energy-dispersive spectrometer (EDS, OXFORD, Oxford, UK) operating at 20 kV. The samples were also characterized by a transmission electron microscope (TEM, FEI Tecnai F30 G2, Hillsboro, OR, USA) operating at an accelerating voltage of 300 kV. The TEM samples were prepared into discs of 3 mm in diameter, mechanically polished to a thickness of 50 μm, and then thinned using a twin-jet electro-polishing method in a solution of 70 vol.% nitric acid and 30 vol.% methanol below −25 °C at 15 V.

## 3. Results

### 3.1. Microstructure of Suction Casting Al–1.8 wt.% Mn Alloy and Al–4 wt.% Mn Alloy

**Figure 1:**
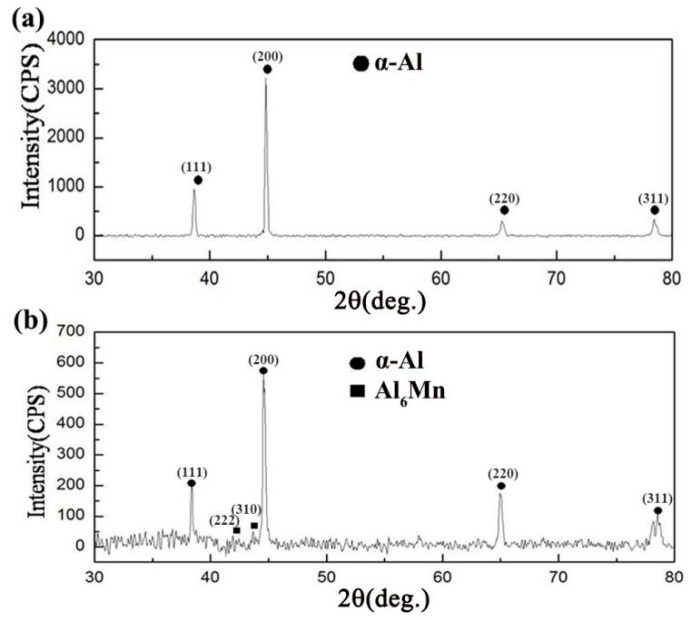
X-ray diffraction (XRD) pattern of suction casting alloys: (**a**) Al–1.8 wt.% Mn alloy; (**b**) Al–4 wt.% Mn alloy.

Figure 1a,b give the XRD patterns of suction casting Al–1.8 wt.% Mn alloy and Al–4 wt.% Mn alloy, respectively. It shows that there are two phases of primary Al_6_Mn phase and α-Al matrix in the Al–4 wt.% Mn alloy, while there is only α-Al matrix in the Al–1.8 wt.% Mn alloy. According to the Al–Mn binary phase diagram [16], it can be seen that the final phase of solidification microstructure in the Al–Mn alloy with Mn content ≥ 2.016 wt.% (eutectic point) is α-Al phase and Al_6_Mn phase, which is consistent with the XRD results.

**Figure 2:**
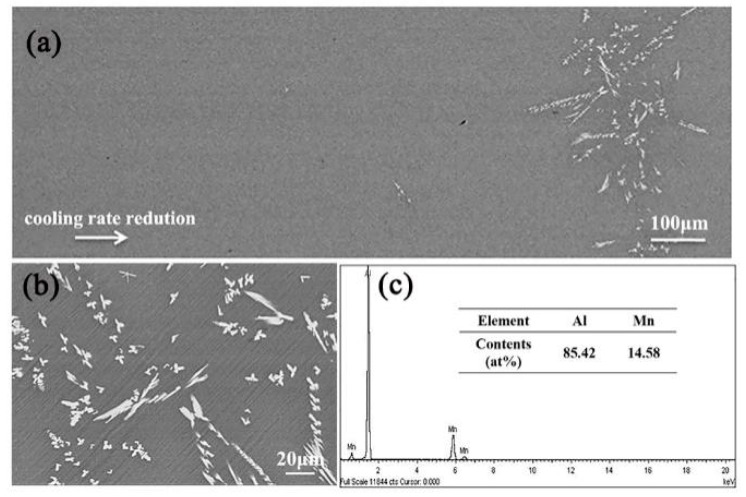
Microstructures of cross-section of the cylindrical sample in suction casting Al–4 wt.% Mn alloy: (**a**) SEM backscattered electron (BSE) image; (**b**) SEM BSE at partial magnification; (**c**) energy-dispersive spectroscopy (EDS).

As observed in the SEM backscattered electron (BSE) image of suction casting Al–4 wt.% Mn alloy in Figure 2a, a small amount of discontinuous block Al_6_Mn crystals occurred at the center of the cylindrical sample. The microstructure of block crystals in Figure 2b consists of sharp edges and corners, while Figure 2c presents the chemical composition of the block phase. The contents of Al and Mn were 85.42 at.% and 14.58 at.%, respectively, with an atomic ratio close to 6:1, which corresponds to the Al_6_Mn phase. The results indicate that only irregular block Al_6_Mn crystals were distributed in the α-Al matrix at the center of the cylindrical sample in suction casting Al–4 wt.% Mn alloy.

### 3.2. Microstructure of Suction Casting Al–6 wt.% Mn Alloy

**Figure 3:**
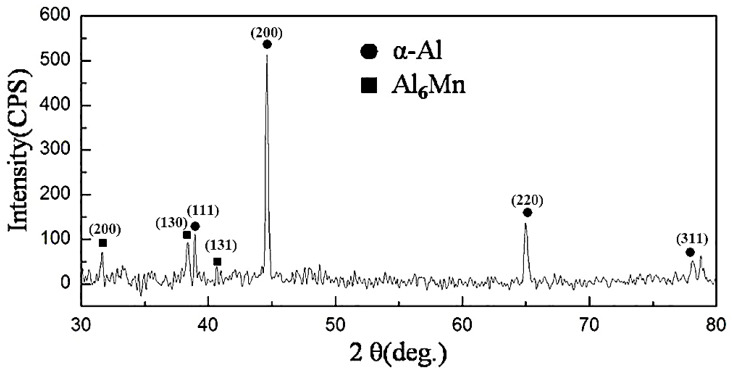
XRD pattern of suction casting Al–6 wt.% Mn alloy.

The XRD pattern of suction casting Al–6 wt.% Mn alloy is shown in Figure 3. There are two types of diffraction peaks in the pattern, namely, α-Al phase and Al_6_Mn phase; thus, it can be inferred that the microstructure of suction casting Al–6 wt.% Mn alloy is composed of α-Al matrix and the primary Al_6_Mn phase.

**Figure 4:**
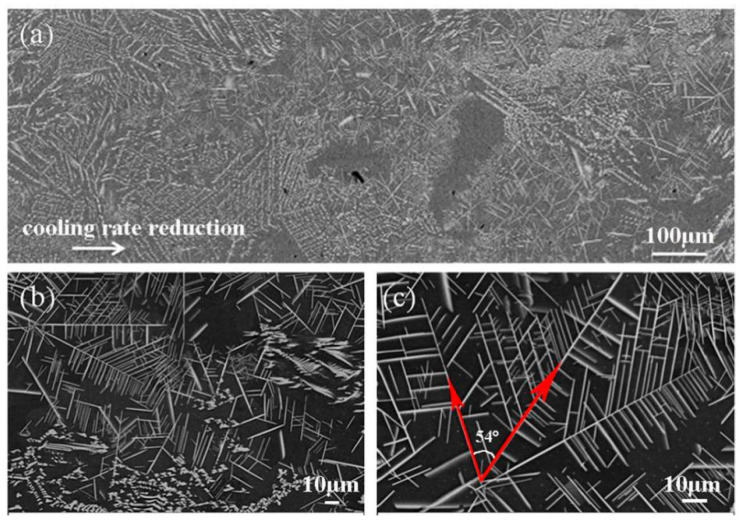
SEM BSE of cross-section of the cylindrical sample in suction casting Al–6 wt.% Mn alloy: (**a**) low magnification; (**b**) high magnification; (**c**) dendrite magnification.

The typical microstructure of the cross-section of the cylindrical sample is displayed in Figure 4a. The black region refers to the α-Al matrix, while the white region reveals the Al_6_Mn phase with different morphologies, which is the same as the result in Reference [25]. The detailed SEM images are shown in Figure 4b,c. Discontinuous block phases are interdigitated with the dendrites on the α-Al matrix, and the block phases are arranged in a disordered fashion, whereas the dendritic phases are present with distinct dendritic stems and secondary dendrite arms. In addition, the block phase has sharp edges and corners, as shown in Figure 4b, which is the same as Figure 2b. In Figure 4c, a dendrite consists of one dendritic trunk and numerous secondary dendrite arms, in which the secondary dendrite arms exhibit a roughly uniform distribution, and they show a constant angle of 54° with the dendrite trunk. The Al_6_Mn phase firstly transforms into a morphology with underdeveloped secondary dendritic arms and finally transforms into the developed secondary dendrites.

**Figure 5:**
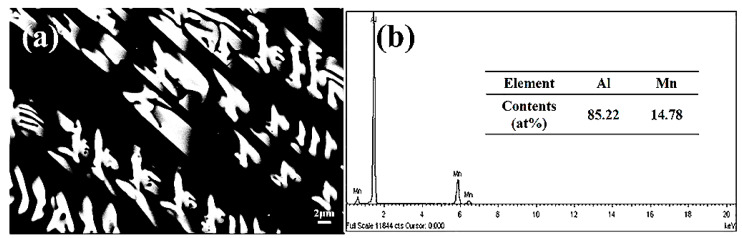
(**a**) Microstructure and (**b**) EDS of block crystals in cross-section of the cylindrical sample.

**Figure 6:**
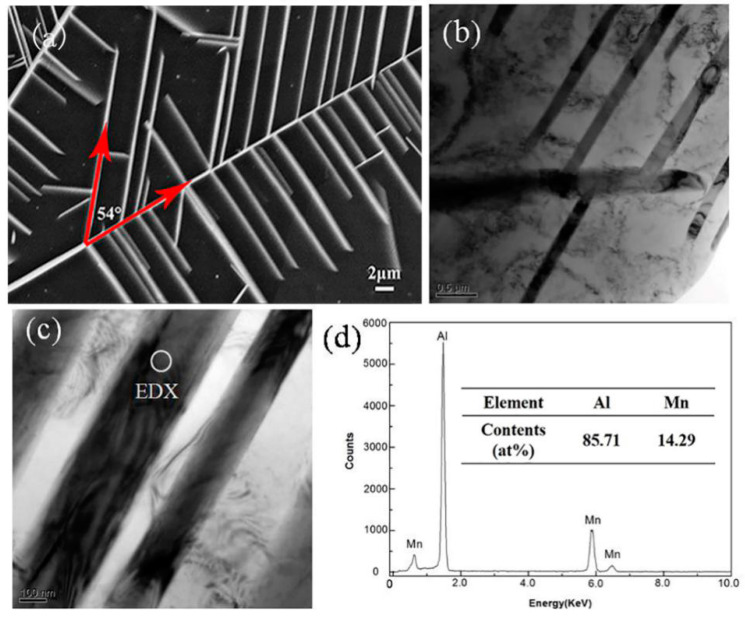
Dendrite microstructure of the cross-section of the cylindrical sample in suction casting Al–6 wt.% Mn alloys: (**a**) SEM BSE; (**b**,**c**) TEM BF (bright field); (**d**) TEM EDS.

Figure 5 is the microstructure and EDS of block crystals in the cross-section of the cylindrical sample. Figure 5b presents the chemical composition of the block phase. The contents of Al and Mn were 85.22 at.% and 14.78 at.%, respectively, with an atomic ratio close to 6:1, which corresponds to the Al_6_Mn phase. Figure 6a displays the SEM BSE image of the cross-section of the cylindrical sample in suction casting Al–6 wt.% Mn alloys. It shows that the microstructure features a feathery dendrite structure with an approximately 54° angle between the primary dendrite and the secondary dendrites, while the secondary dendrite arm spacing is roughly estimated to be 4 μm. In order to reveal more details about the morphological features, TEM bright-field (BF) imaging was used, as shown in Figure 6b,c. The dendritic structure is shown in high contrast, while the interface has no obvious diffusion layer, which suggests that the phase has strict stoichiometric compounds. The chemical composition of the phase was analyzed by EDS, as listed in Figure 6d. The dendrite chemical composition was 85.71 at.% Al and 14.29 at.% Mn, with an atomic ratio of 6:1. Therefore, dendrite crystals were in the Al_6_Mn phase. From the SEM EDS and TEM EDS, it can be deduced that the dendrite and bulk crystals were the two different morphologies of the Al_6_Mn phase.

### 3.3. Microstructure of Suction Casting Al–8 wt.% Mn Alloy

**Figure 7:**
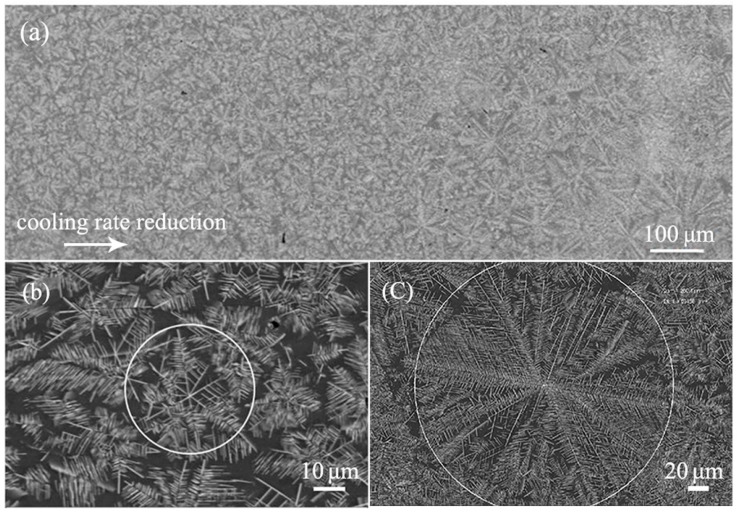
SEM BSE of the cross-section of the cylindrical sample in suction casting Al–8 wt.% Mn alloy: (**a**) full sample; (**b**) at the edge of the sample; (**c**) at the center of the sample.

Figure 7a shows the SEM BSE of the cross-section of the cylindrical sample in suction casting Al–8 wt.% Mn alloy. The α-Al matrix is covered with dendrites, which have well-developed primary and secondary arms. From the edge to the center of the sample, the outline of the equiaxed dendrite gradually increases due to the drop in the cooling rate. The cooling rate at the center of the sample is relatively slow such that the dendrite has a longer time to grow. Figure 7b gives a detailed image of the dendrite at the edge of the sample. It can be seen that about eight dendrite trunks grow from a nucleation particle on the observation surface, and each dendrite trunk has its own secondary dendrite arms. The average outline diameter of the equiaxed dendrite is equal to 43 μm. Figure 7c displays the microstructure of the center of the sample. Compared with the edge of the sample, the dendrite trunk in this position is more developed, but the secondary dendrite arms become relatively short. The outline diameter of the equiaxed dendrite is about 275 μm, which is more than six times that of the equiaxed dendrite diameter at the edge of the sample.

**Figure 8:**
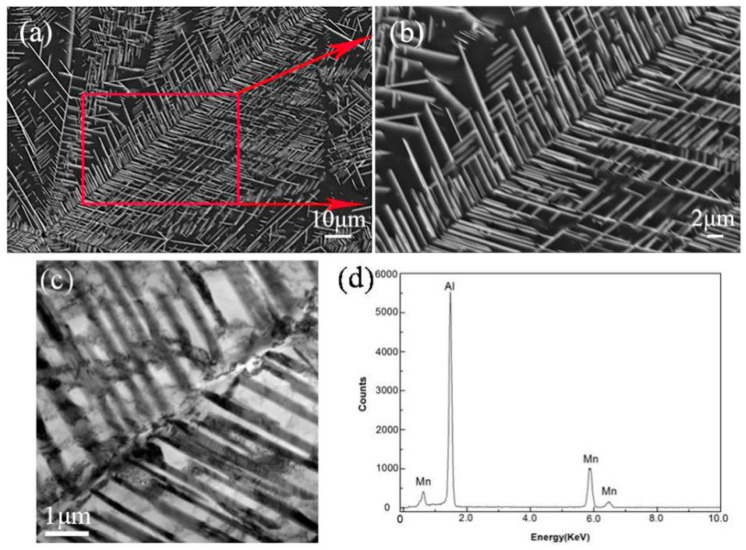
Dendrite microstructure at the center of the cross-section of the cylindrical sample in suction casting Al–8 wt.% Mn alloys: (**a**) SEM BSE; (**b**) SEM BSE; (**c**) TEM BF; (**d**) TEM EDS.

**Figure 9:**
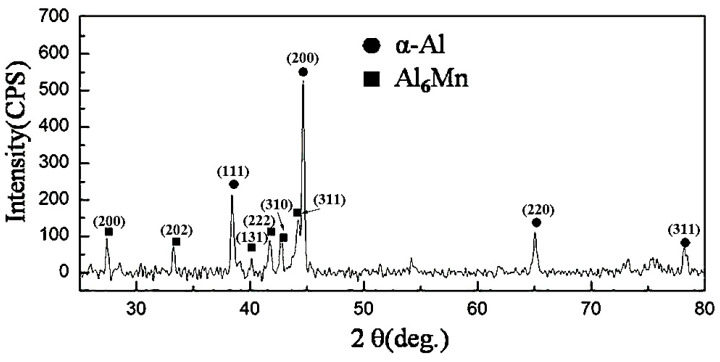
XRD pattern of suction casting Al–8 wt.% Mn alloy.

Detailed information on the microstructure at the center of the sample is provided in Figure 8. The dendrites were identified by combining SEM, TEM BF imaging, and TEM EDS. It is obvious that the dendrite trunks exhibit a snowflake in the eutectic matrix. The primary dendrites are very developed with an interdendritic region, and secondary dendrite arms farther from the nucleation point are longer. The microstructure formed because of a mutual inhibition along the adjacent primary dendrites. Thus, the secondary dendritic arms away from the nucleation particle had more space to grow. The higher-magnification images in Figure 8b reveal that the secondary dendritic arms were aligned at an angle of 72° along the trunk stem. The fine details of the microstructure of the dendrite were studied using TEM, and the results are given in Figure 8c,d. The secondary dendrite arm spacings were about 1 μm. According to the EDS analysis result in Figure 8d, it can be seen that the elemental content of the dendritic trunk and the secondary dendrite arm was almost the same, with an atomic ratio of Al to Mn close to 6:1; thus, it can be confirmed that the dendrite was also the Al_6_Mn compound phase. According to the XRD pattern of suction casting Al–8 wt.% Mn alloy shown in Figure 9, the alloy has only two phases of α-Al and Al_6_Mn, which is consistent with the microstructure and EDS composition analysis results. Therefore, there is only the dendritic Al_6_Mn compound phase distributed on the α-Al matrix in suction casting Al–8 wt.% Mn alloys.

### 3.4. Microstructure of Suction Casting Al–10 wt.% Mn Alloy

**Figure 10:**
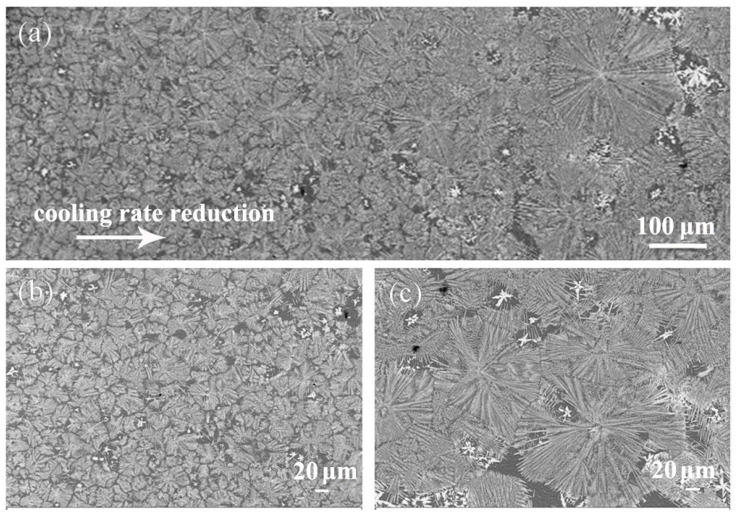
SEM BSE of the cross-section of the cylindrical sample in suction casting Al–10 wt.% Mn alloy: (**a**) full sample; (**b**) at the edge of the sample; (**c**) at the center of the sample.

The microstructure of the cross-section of the cylindrical sample in suction casting Al–10 wt.% Mn alloy is shown in Figure 10a. It is clearly seen that the matrix is covered with a lotus leaf-like phase, which is similar to the microstructure in the Al–8 wt.% Mn alloy, but the dendrite trunks and secondary dendrite arms are more developed. Therefore, it can be judged that the dendrite is an Al_6_Mn compound phase. In addition, there is a bright petal-like phase distributed between the developed dendrites, which were never found in vacuum suction casting alloys with Mn content less than 10 wt.%. The evolution of the dendrite is shown in Figure 10b,c as the cooling rate decreases. As demonstrated in Figure 10b,c, the outline diameter of the equiaxed dendrite became smaller at the edge with increased cooling rate. This is mainly because a larger cooling rate results in more nucleation sites of the alloy and, thus, more dendrites formed.

**Figure 11:**
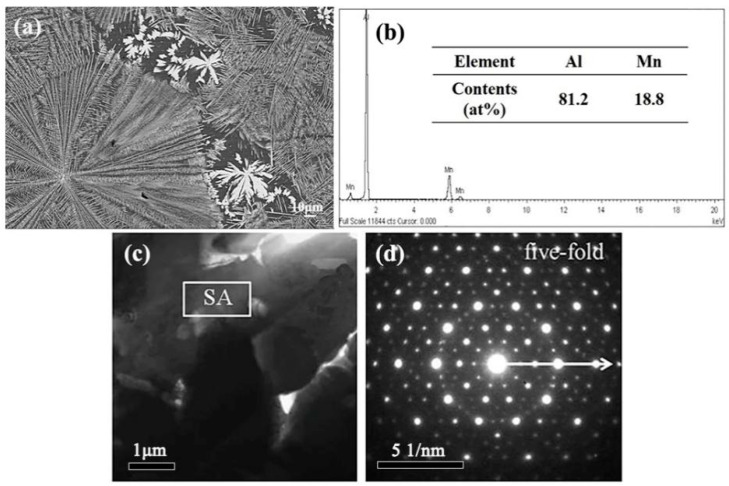
Microstructure of the cross-section of the cylindrical sample in suction casting Al–10 wt.% Mn alloys: (**a**) SEM BSE; (**b**) EDS; (**c**) TEM BF; (**d**) selected-area electron diffraction (SAED).

**Figure 12:**
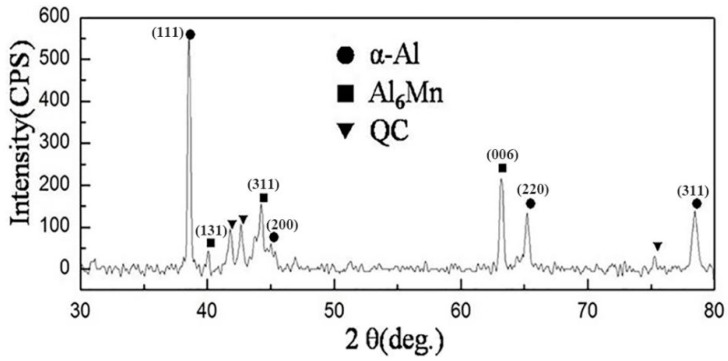
XRD pattern of suction casting Al–10 wt.% Mn alloy.

Figure 11a shows the microstructure of the dendritic and petal-like phases in the Al–10 wt.% Mn alloy. The dendritic region refers to the Al_6_Mn phase, which is consistent with the Al–8 wt.% Mn alloy. The petal-like phase is similar to the phase in the Al–6 at.% Mn alloy by Shechtman et al. [4] obtained by rapid solidification, which was identified as a typical icosahedral quasicrystal morphology. In order to determine whether it was an icosahedral quasicrystal, SEM EDS was used. From the result in Figure 11b, in the phase with an atomic content of 81.2 at.% Al and 18.8 at.% Mn, the atomic ratio was very close to that of the Al_4_Mn phase, which is a quasicrystal-approximating phase with a hexagonal crystal structure [26]. In fact, not only was the element distribution similar to the quasicrystal, but the local atom clusters were also arranged in the same way as the atoms in the quasicrystal formation. Therefore, from the elemental composition, it was impossible to distinguish the Al_4_Mn phase and the quasicrystal phase, and it was also impossible to determine whether the petal-like morphology of the Al–10 wt.% Mn alloy was an icosahedral quasicrystal phase. Thus, the petal-like phases were further characterized by TEM and corresponding selected-area electron diffraction (SAED), and the results are presented in Figure 11c,d. Figure 11c shows the BF image of the petal-like phase, and the corresponding SAED pattern of the phase is indicated in Figure 11d. It is obvious that the petal-like phase shows a five-fold symmetry. It can be confirmed as the icosahedral quasicrystal phase because of its unique rotational symmetry, which is different from the traditional crystal rotational symmetry [27,28]. Therefore, it can be determined that the petal-like phase is a quasicrystalline phase. Combined with the XRD result in Figure 12, it can be concluded that the microstructure of suction casting Al–10 wt.% Mn alloy is composed of α-Al, dendritic Al_6_Mn phases, and petal-like quasicrystal phases.

## 4. Discussion

As we all know, the growth morphologies of crystals are mainly determined by the crystal structure and external growth conditions [29]. The inherent structure of the crystal causes the crystal to grow toward the equilibrium morphology with the smallest surface free energy, while the external growth conditions lead to the crystal deviating from equilibrium and becoming other diverse appearances. Solute flux, heat flow, and competitive growth between adjacent crystals are important external factors that affect the non-equilibrium crystal growth [30]. As can be seen from previous studies [10,25], it can be shown that, with an increase in the solute Mn content, the morphology of the Al_6_Mn compound phase in vacuum suction casting Al–Mn alloys changes greatly. In fact, the vacuum suction casting cylindrical copper mold has a great influence on the heat flow and solute transport of the melt [31]. The heat flow is transmitted outward from the center of the cross-section of the specimen along the radius. Firstly, intermittent block crystals are formed under low-manganese conditions, while dendrites with different degrees of development are formed under high-manganese conditions. Then, the dendrite morphology of the Al_6_Mn compound phase at the same manganese content was converted from an undeveloped shape to a highly developed branch shape with an increase in cooling rate. The dendritic growth behavior of the Al_6_Mn phase in vacuum suction casting alloys can be determined based on both the solute element content and the cooling rate. This conclusion was also obtained from the latest two papers of Stan-Głowińska [19,32]. He evaluated the effects of Fe, Cr, Co, Ni, and Cu addition on quasicrystalline phase formability in 94Al–6Mn base alloys obtained by wedge casting. In the case of the addition of different elements, the quasicrystalline phase thickness of the prepared casting depended on the cooling rates present in different parts of the sample.

**Figure 13:**
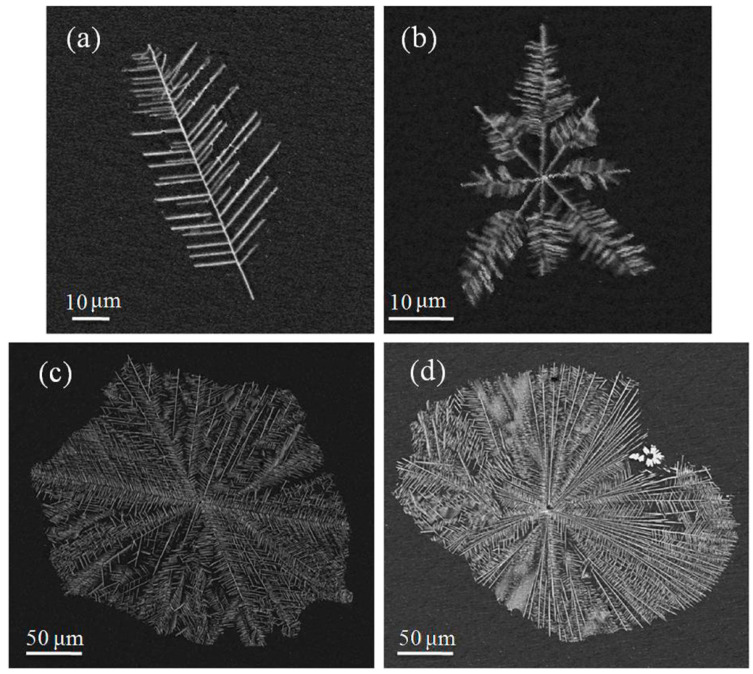
SEM BSE morphology of Al_6_Mn dendrites in suction casting Al–Mn alloys: (**a**) Al–6 wt.% Mn alloy; (**b**) at the edge of the cross-section of the sample in the Al–8 wt.% Mn alloy; (**c**) at the center of the cross-section of the sample in the Al–8 wt.% Mn alloy; (**d**) at the center of the cross-section of the sample in the Al–10 wt.% Mn alloy.

Figure 13 shows the different morphologies of the Al_6_Mn dendrites in vacuum suction casting Al–Mn alloys, all of which were complete dendrites obtained under SEM BSE mode. As shown in Figure 13a of the Al–6 wt.% Mn alloy, there was only one trunk in the dendrites, and the secondary arms of the dendrites were aligned parallel to the dendrite trunk at 54°. This microstructure was firstly transformed from a solid polygonal shape. During the solidification of the Al–Mn alloy, the Al_6_Mn phase precipitated out of the liquid phase and exhibited anisotropic growth with sharp edges and corners. The crystal tended to be spilt as the cooling rate increased, and it finally became a branch shape during growth. Based on the solidification theory [33], when the front of the liquid–solid interface does not produce composition undercooling, the interface growth is planar. On the contrary, if there is composition undercooling in the liquid–solid interface, the interface will lose its stability and grow in a cell shape or a dendritic shape. When the solute Mn content increased to 8 wt.%, the dendrite Al_6_Mn phase was covered with the α-Al matrix. Compared with the Al–6 wt.% Mn, the block morphology disappeared in the Al–8 wt.% Mn alloy, whereas they both showed the morphology of dendrites. As shown in Figure 13b at the edge of the cross-section of the sample, the dendrite morphology was much more complex than that of Figure 13a. Multiple dendritic trunks grew from the nucleation particle on each side. Each of the dendritic trunks had secondary arms similar to those in Figure 13a. According to the diffusion limit aggression proposed by Witten and Sander [34], the snowflake growth is mainly controlled by the diffusion process, in which the surrounding growth points diffuse toward the center and adhere to the growth points. Any particle that can diffuse into a fractal can grow continuously, thus forming a branch-like epitaxy. Since the dendrite in Figure 13b was located at the edge of the sample with a ~10^3^ K/s cooling rate, where the cooling rate was faster and the nucleation rate was higher, the dendrite was less able to grow sufficiently. Therefore, the dendrite at this location was small in outline size, and the dendrite outline diameter was only 43 μm. The dendrite in Figure 13c was near the center of the sample with a ~10^2^ K/s cooling rate, where the cooling rate was slower than that of the edge, and a large number of dendritic stems grew from the nucleation particle before becoming bulky and lush. The secondary arms then began to shrink and became shorter. The outline diameter of the entire dendrite reached an astonishing 275 μm. The shape of the dendrite appeared regular and close to a circle. It can be inferred that the nucleation was more uniform here. When the solute Mn content was increased to 10 wt.%, the dendrite near the center of the sample became more developed. As shown in Figure 13d, the number of dendrite trunks further increased, and the dendritic secondary arms began to degenerate. The shape of the trunk was not very regular, and the dendritic trunk varied in length. There were two factors that caused the length change of the dendrite trunk in Figure 13d, that is, the mutual inhibition between the growth of adjacent dendrites and the growth delay of some dendrite trunks because of the appearance of quasicrystals.

For Al–Mn alloys, a fast cooling rate reduces the solute content required for quasicrystal formation. In this experiment, the minimum Mn content of quasicrystals formed by the vacuum suction casting method reached 10 wt.%. The interfacial energy between the icosahedral quasicrystal phase and the liquid phase was small; thus, so the activation energy required to form the nucleus of the quasicrystal phase was also small [35]. Therefore, quasicrystals are often considered to preferentially nucleate from the liquid phase. During solidification, the icosahedral quasicrystalline phase was first nucleated and grown from the liquid phase, and it was retained with rapid solidification, while the post-nucleated Al_6_Mn phase was retained. In the process of dendritic growth, some dendritic trunks encounter quasicrystalline phases and stop growing. Some dendritic trunks continue to grow without encountering quasicrystalline phases until they are hindered by neighboring dendrites and stop growing. This explains the formation of dendrites in Figure 13d.

## 5. Conclusions

In this work, the effects of solute element content and cooling rate on the microstructure of binary Al–Mn alloys under suction casting were investigated. The main conclusions are as follows:

(1) The microstructure in suction casting Al–Mn alloys at higher Mn content (10 wt.%) is composed of Al_6_Mn dendrites, Al_4_Mn quasicrystals, and α-Al, while that at lower Mn content (4 wt.%, 6 wt.%, and 8 wt.%) is only composed of Al_6_Mn phases and α-Al. The Mn content has a positive effect on improving the formation of quasicrystals.
(2) With the increase in solute Mn content, the morphology of Al_6_Mn dendrites changed from a block to a feathery phase, and then to snowflake-like and the lotus leaf-like phases. The outline diameter of equiaxed dendrite decreased with the increase in cooling rate from the center to the edge of samples.
(3) The formation of icosahedral quasicrystals can delay the growth of some dendrite trunks in an equiaxed dendrite.
